# Cytology cell blocks from malignant pleural effusion are good candidates for PD-L1 detection in advanced NSCLC compared with matched histology samples

**DOI:** 10.1186/s12885-020-06851-z

**Published:** 2020-04-22

**Authors:** Yinying Zou, Liming Xu, Qiusu Tang, Qihan You, Xiaoling Wang, Wei Ding, Jing Zhao, Guoping Ren

**Affiliations:** 1 Department of Pathology, The First Affiliated Hospital, College of Medicine, Zhejiang University, No. 79, Qingchun Road, Xiacheng District, Hangzhou, 310003 China; 2 Department of Pathology and Pathophysiology, School of Medicine, Zhejiang University, Hangzhou, 310058 China

**Keywords:** Cytology, Immunohistochemistry, Malignant pleural effusion, Non-small-cell lung carcinomas, PD-L1

## Abstract

**Background:**

Detection of programmed cell death ligand-1 (PD-L1) by immunohistochemistry (IHC) has been commonly used to predict the efficacy of treatment with PD-1/PD-L1 inhibitors. However, there is limited literature regarding the reliability of PD-L1 testing using malignant pleural effusion (MPE) cell blocks. Here, we assess PD-L1 expression in sections from MPE cell blocks and evaluate the value of IHC double staining in the interpretation of PD-L1 expression.

**Methods:**

In all, 124 paired formalin-fixed tissues from advanced NSCLC patients, including MPE cell blocks and matched histology samples, were included. PD-L1 expression was assessed using the SP263 assay, and the tumor proportion score (TPS) and the staining intensity were evaluated. PD-L1 staining results were also compared between IHC double and single staining techniques.

**Results:**

PD-L1 expression was concordant in most paired cases (86/101, 85.1%) among three TPS cut-offs (<1%, 1–49% and ≥ 50%), with a kappa value of 0.774. Moreover, a significant difference in PD-L1 expression between MPE cell blocks and biopsy samples was observed (*p* = 0.005). For the 15 discordant pairs, 13 MPE cell block samples showed increased expression of PD-L1. Compared with the standard IHC single PD-L1 assay, double staining with anti-TTF-1 and anti-PD-L1 revealed a negative effect on PD-L1 expression testing and resulted in weaker staining intensity and a lower TPS (*p* = 0.000).

**Conclusions:**

MPE cell block samples are good candidates for PD-L1 expression detection in advanced NSCLC patients. The mechanism and clinical significance of the higher PD-L1 expression rate in MPE cell blocks compared with small biopsy samples remain to be evaluated prospectively.

## Background

For the past few years, immune checkpoint inhibitors that inhibit the interaction between programmed cell death protein-1 (PD-1) and its tissue ligand programmed cell death ligand-1 (PD-L1) have shown active and enduring clinical efficacy in a variety of solid tumors, including non-small cell lung carcinoma (NSCLC) [1–4]. However, considering the potential immune-related adverse reactions and high cost, identifying patients who may respond to anti-PD-1/PD-L1 treatment is particularly important. According to recent data, high levels of tumor PD-L1 expression usually indicate an enhanced likelihood that a patient will benefit from treatment with PD-1/PD-L1 inhibitors, [2, 5, 6] nonetheless, a subset of patients negative for PD-L1 expression can still respond to treatment [1, 3]. Although tumor PD-L1 expression is not a perfect biomarker with regard to its ability to predict the efficacy of immunotherapy, PD-L1 detection by immunohistochemistry (IHC) is currently the most convenient and economical method, and it has commonly been used to identify patients who may be more likely to benefit from immunotherapy with PD-1/PD-L1 inhibitors.

As the adverse effects of immunotherapy such as hyperprogressive disease [7–9] are better understood, screening of potentially beneficial populations is becoming increasingly important. Unfortunately, many patients with NSCLC are diagnosed at an advanced stage and sufficient samples are not available for PD-L1 detection because these patients cannot benefit from surgery; sometimes, they cannot tolerate even small biopsies. Malignant pleural effusion (MPE) is a common complication of NSCLC, especially in adenocarcinoma (AC). MPE samples are easy to acquire, and this type of cell block sample is often used as an alternative or sometimes as the only adequate sample for various relevant tests, such as IHC and molecular testing related to targeted therapy [10, 11]. In the past few years, studies on the applicability of small biopsies and cytology samples for PD-L1 IHC detection have become very common [12–18]. To the best of our knowledge, only few data have been published on the reliability of MPE cell blocks for PD-L1 testing; these studies are limited because of the small sample size of the MPE cell blocks [19, 20] or the lack of matched experiments [21, 22]. Due to the heterogeneity of PD-L1 expression by tumor cells, assessing the reliability and comparability of PD-L1 testing on MPE cell blocks by a comparison study is necessary.

As mentioned in many studies, PD-L1 evaluation is indeed a challenge for pathologists because it is sometimes difficult to distinguish tumor cells from immune cells, and this challenge is even more pronounced in cytology samples [23, 24]. To our knowledge, it has not yet been reported whether IHC double staining for nuclear proteins such as thyroid transcription factor-1 (TTF-1) and PD-L1 can resolve this issue.

Herein, the specificity of PD-L1 expression on MPE tumor cells is explored by comparison with matched histology samples, and the relationship between clinicopathological features and tumor PD-L1 expression is also analyzed. Furthermore, IHC double staining for TTF-1 and PD-L1 was also performed in this study, and its application value for interpretation of PD-L1 tumor cell staining was evaluated.

## Methods

### Cases

We retrospectively searched the electronic database of the Department of Pathology, the First Affiliated Hospital of Zhejiang University, Hangzhou, China, for data on MPE cell block samples obtained between January 01, 2015, and December 31, 2017. In all, 124 pairs of MPE cell blocks with matching histology specimens from patients diagnosed with NSCLC were included in the study.

### Specimen preparation

The histology specimens were obtained by surgical resection or small biopsy. After fixation in 10% neutral-buffered formalin (10% NBF), samples were transferred to a Leica ASP300S Fully Enclosed Tissue Processor (Leica Biosystems, Buffalo Grove, IL, USA) for paraffin embedding.

The MPE cell blocks were prepared once malignant cells were confirmed by cytodiagnosis. Briefly, 200–800 ml of fresh pleural effusion fluid was centrifuged at 1600 x g for 3 min. The target cells, which were sampled from the surface of the nucleated cell layer, were then transferred into a pointed centrifuge tube containing 10 ml 10% NBF and oscillated for at least 15 min. Next, the samples were centrifuged again to remove the fixative and were then resuspended in 75% alcohol. Subsequently, a third centrifugation was performed to replace 75% alcohol with 95% alcohol; the material was treated gently to keep the cell mass intact. Afterwards, the samples remained immobile for more than 3 h, until the cell mass hardened and contracted. Finally, well-formed cell pellets were transferred to a dehydrator for final processing with the same procedure used for the histology tissues. All the samples were cut consecutively into 3-μm-thick sections for hematoxylin and eosin (H&E) staining and IHC analysis.

### IHC quantification of PD-L1 expression and evaluation

Quantification of PD-L1 expression was performed using a Ventana PD-L1 (SP263) Rabbit Monoclonal Primary Antibody assay according to the manufacturer’s instructions. The tumor proportion score (TPS) was evaluated by a qualified pathologist who was trained in scoring PD-L1 expression. Membrane staining (local/global) at any intensity greater than background staining was evaluated, and only viable tumor cells (VTCs) were scored. Cases with too few VTCs (<100) were considered inadequate and were not included. PD-L1 expression was finally divided into three categories according to the TPS: < 1% (negative), 1–49% (low expression) and ≥ 50% (high expression). The staining intensity score (SIS) of PD-L1 expression was also recorded, as follows: 3+ (strong), 2+ (moderate), and 1+ (weak). A subset of paired samples with discrepant results in terms of PD-L1 expression was repeatedly tested, and the results were interpreted by an additional pathologist. The expression pattern of TTF-1 from IHC double-stained sections was taken as reference if necessary.

### IHC double staining with PD-L1 and TTF-1

Positive cases with inconsistent PD-L1 expression in the paired samples (excluding 2 MPE cell blocks and 4 biopsy samples with insufficient remaining material), as well as other difficult-to-interpreted samples, were sectioned again for IHC double staining with anti-TTF-1 (clone SPT24, OriGene, USA) and anti-PD-L1 (clone SP263). In all, 32 samples (20 MPE cell blocks and 12 histology samples) from 18 lung AC patients with positive PD-L1 expression were enrolled.

Automated Ventana IHC analysis for PD-L1 and TTF-1 was successively performed on the same slide according to the manufacturer’s instructions for each antibody. The PD-L1 detection procedure was first performed according to the previous single staining process, which was followed by a subsequent automated Ventana IHC analysis for TTF-1 (dilution 1:100) using the same Ventana Benchmark ULTRA staining platform with an ultraView Universal AP Red Detection Kit (Ventana Medical Systems, Tucson, AZ, USA). The external positive/negative controls for PD-L1- and H&E-stained slides were also established.

### Statistical analysis

The consistency of PD-L1 IHC expression among matched samples was analyzed by calculating the kappa coefficient (weak consistency for kappa value<0.4, moderate for kappa value = 0.4–0.74 and good for kappa value ≥0.75), and their difference was compared using the marginal homogeneity test. Pearson’s chi-square test or Fisher’s exact test was used to compare the consistency of the PD-L1 expression rate between matched samples according to different factors (sample interval time, treatment, sampling method of histology samples, and number of VTCs). Fisher’s exact test was also employed to compare the satisfaction rate among histology samples with different sampling methods, while the McNemar-Bowker test was used when MPE cell blocks and matched histology specimens were compared between two groups. The Mann-Whitney U-test was performed to explore the correlation between the PD-L1 expression in patients with their characteristics (sex, age, smoking status, and clinicopathological diagnosis). The Kruskal-Wallis test was applied to compare PD-L1 expression in histology samples for which different sampling methods were used. All the data were analyzed using SPSS 24.0 (IBM Corporation, Armonk, NY, USA), and the two-sided significance level was set at *p* < 0.05.

## Results

### Clinicopathological features of patients and specimens

In all, 124 paired NSCLC specimens were collected from 124 patients with a median age of 61 years (range, 29–85 years) (Table 1). Most of the samples were satisfactory, and the adequacy of paired specimens was similar (91.1% vs 89.5%). For histology specimens, the adequacy of various sampling methods was slightly different, but the difference was not statistically significant.

**Table 1:** Clinical and pathologic details of patients and specimens

Characteristic	No.	Adequate (%)
Specimens	248	225 (90.7)
MPE cell blocks	124	114 (91.9)
Histology specimens	124	111 (89.5)
Site		
Lung	75	
Regional/distant lymph nodes	29	
Thorax/mediastinum	16	
Bone	2	
Subaxillary/abdominal mass	2	
Type		
Surgical resection	11	11 (100)
EBUS-TBNA	8	8 (100)
Biopsy	105	92 (87.6)
Endobronchial forceps biopsy	38	30 (78.9)
CTG-CN	31	30 (96.8)
Other histologic biopsy	36	32 (88.9)
Patients	124	
Age (median) (y)	29–85 (61)	
Sex		
Male	80	
Female	44	
Diagnosis		
Adenocarcinoma	108	
Squamous cell	11	
NSCLC, NOS	5	

Abbreviations: *MPE* malignant pleural effusion, *CTG-CN* computed tomography-guided core needle biopsy, *EBUS-TBNA* endobronchial ultrasound-guided transbronchial needle aspiration biopsy, *NSCLC, NOS* non-small cell lung carcinoma, not otherwise specified

### PD-L1 expression in matched specimens

Excluding 23 unsatisfactory cases, 101 paired samples were successfully analyzed. Expression of PD-L1 was concordant in most cases (86/101, 85.1%) among the three TPS cut-offs (<1%, 1–49% and ≥ 50%), and the consistency of PD-L1 expression between MPE cell blocks and matched histology samples was confirmed by the kappa test (kappa = 0.774, *p* = 0.000<0.05). However, compared with matched histology samples, MPE cell blocks had a higher rate of positive PD-L1 expression (39.6% vs 30.7% with TPS ≥50%), and the major difference was a focus on biopsy and corresponding MPE cell block samples (*p* = 0.005<0.05) (Table 2). PD-L1 expression was discordant in 15 paired cases, which were all ACs (Table 3). Among them, PD-L1 expression was higher in 2 histology specimens and 13 MPE cell blocks compared with their corresponding samples.

**Table 2:** PD-L1 expression in MPE cell blocks and matched histology samples

Type	Tumor proportion score of PD-L1			Total (n)	Coincidence rate (%)	*P* value
	< 1%, n (%)	1–49%, n (%)	≥50%, n (%)			
MPE cell block	23 (22.8)	38 (37.6)	40 (39.6)	101	85.1	0.005^a^
Histology sample	26 (25.7)	44 (43.6)	31 (30.7)	101		
Surgical resection	3 (27.3)	5 (45.5)	3 (27.3)	11	72.7	0.564
Biopsy	20 (24.1)	38 (45.8)	25 (30.1)	83	85.5	0.005^a^
EBUS-TBNA	3 (42.9)	1 (14.3)	3 (42.9)	7	100	1.000

Abbreviations: *PD-L1* programmed cell death ligand-1, *MPE* malignant pleural effusion; EBUS-TBNA, endobronchial ultrasound-guided transbronchial needle aspiration biopsy; ^a^statistically significant

**Table 3:** Detailed information of discordant cases for PD-L1 expression among paired samples

No.	Specimen 1					Time interval	Specimen 2				
	Type	VTC	Treatment	Staining intensity	PD-L1%		Type	VTC	Treatment	Staining intensity	PD-L1%
1	Surgical resection (lung)	10,000	NT	3+	70	18.5 m	MPE	10,000	AIT^a^	3+	40
2	Subaxillary mass biopsy	790	CM, AIT	1+	15	10.2 m	MPE	10,000	CM^a^, AIT	3+	90
3	CTG-CN (lung)	400	NT	1+	45	5.1 m	MPE	100,000	TGT^a^	3+	90
4	CTG-CN (lung)	5000	NT	1+	< 1	1.6 m	MPE	10,000	CM^a^, EBRT^a^	3+	> 90
5	Surgical resection (LN)	30,000	NT	1+	15	3 d	MPE	56,000	NT	2+	70
6	MPE	320	CM	1+	20	0 d	Pleural biopsy	240	CM	2+	70
7	MPE	5000	NT	2+	50	0 d	Surgical resection (LN)	10,000	NT	2+	5
8	MPE	6000	NT	2+	55	0 d	Endobronchial FB	450	NT	1+	15
9	MPE	150,000	NT	3+	> 90	1 d	Endobronchial FB	234,000	NT	1+	10
10	MPE	80,000	NT	3+	> 90	2 d	CTG-CN (lung)	37,100	NT	2+	20
11	MPE	500	NT	3+	> 90	2 d	Endobronchial FB	400	NT	1+	40
12	MPE	30,000	NT	3+	> 90	4 d	Pleural biopsy	1060	NT	2+	35
13	MPE	125	NT	2+	20	6 d	Pleural biopsy	450	NT	1+	< 1
14	MPE	10,000	NT	3+	90	10 d	Lymph node biopsy	550	NT	1+	2
15	MPE	1540	NT	1+	20	19.1 m	Pleural biopsy	4520	TGT^a^	1+	< 1

Abbreviation: *VTC* viable tumor cells, *MPE* malignant pleural effusion from thoracentesis, *PD-L1* programmed cell death ligand-1, *CTG-CN* computed tomography-guided core needle biopsy, *FB* forceps biopsy, *NT* no treatment, *CM* chemotherapy, *TGT* targeted therapy, *AIT* adoptive cellular immunotherapy, *EBRT* external beam radiotherapy; ^a^different from previous treatments; Specimen 1 obtained earlier than specimen 2

**Fig. 1:**
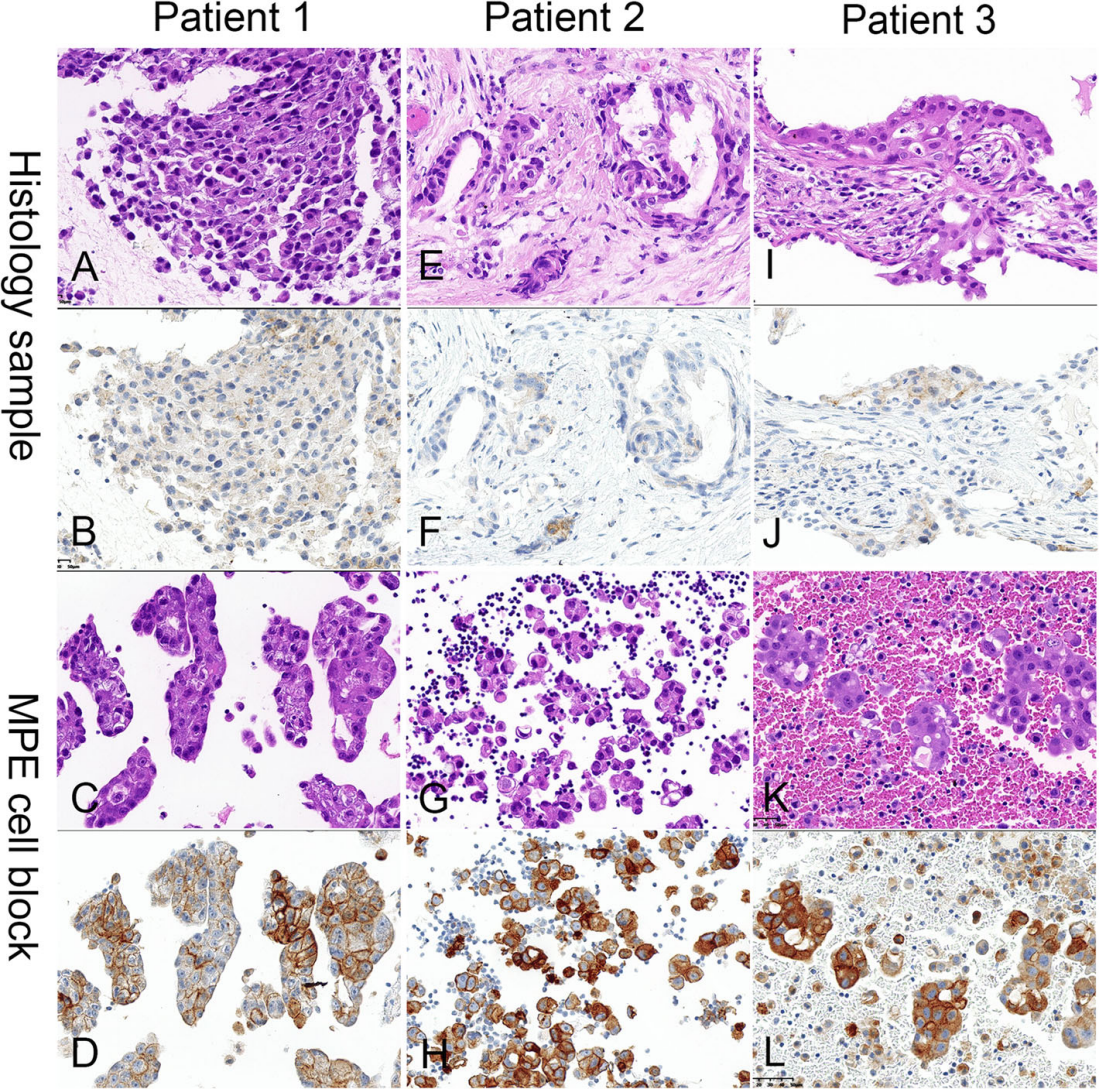
Inconsistent PD-L1 expression in matched MPE cell block and histology samples from three NSCLC patients (magnification × 40). Representative images of hematoxylin–eosin staining for a lymph node biopsy (**a**), a pleural biopsy (**e**), a CTG-CN biopsy (**i**) and the matched MPE cell blocks (**c, g, k**). PD-L1 IHC (SP263) shows higher PD-L1 expression and/or stronger staining intensity in MPE cell blocks compared with matched histology samples (**d** vs **b**; **h** vs **f**; **l** vs **j**). PD-L1, programmed cell death ligand-1; MPE, malignant pleural effusion; CTG-CN, computed tomography-guided core needle biopsy

By comparing the SIS of PD-L1 expression in matched samples, we found that the intensity of PD-L1 positive staining in MPE specimens was often stronger than that in corresponding histology samples (*p* = 0.000<0.05) (Fig. 1) and that the coincidence rate was 73.3%. However, after excluding the 15 pairs of discordant cases, the difference was not significant (*p* = 0.074) (Table 4).

**Table 4:** Consistency of staining intensity scores for the PD-L1 IHC assay between paired samples

Type	PD-L1 staining intensity scoring				Total (n)
	0	1+	2+	3+	
MPE cell blocks	23	23 + 2^a^	17 + 4^a^	20 + 9^a^	83 + 15^a^
Histology samples	23	28 + 10^a^	15 + 4^a^	17 + 1^a^	83 + 15^a^

Abbreviation: *PD-L1* programmed cell death ligand-1, *MPE* malignant pleural effusion; ^a^samples with discordant tumor proportion scores

### Correlation between PD-L1 expression and various factors

In this study, if PD-L1 expression was inconsistent between the matched samples in one patient, the higher score was considered the final result. Compared with squamous cell carcinoma (SCC) and positive smoking status, patients with AC or nonsmoking status had higher tumor PD-L1 expression rates (*p* = 0.013 and 0.009). Considering the association between nonsmoking status and lung AC, we separated lung cancers into two independent groups (AC and SCC) and re-studied the relationship between the expression of tumor PD-L1 and smoking status in each group. We observed a possible trend toward statistical significance in AC subgroup (*p* = 0.052) (Table 5). When 1% was used as the threshold for TPS, smokers in the AC subgroup were more likely to have negative expression (TPS<1%) of PD-L1 (*p* = 0.043<0.05). No significant difference was observed in PD-L1 expression among samples obtained from different tissue sources or by different sampling methods.

**Table 5:** PD-L1 expression stratified by clinicopathologic features

Characteristic	PD-L1 TPS			Total (n)	Z	*P* value
	< 1% n (%)	1–49% n (%)	≥50% n (%)			
Total	32 (25.8)	45 (36.3)	47 (37.9)	124		
Age, y				124	−1.038	0.299
≤ 55	13 (29.5)	17 (38.6)	14 (31.8)			
> 55	19 (23.8)	28 (35)	33 (41.3)			
Sex				124	−1.099	0.272
Male	23 (28.8)	29 (36.3)	28 (35.0)			
Female	9 (20.5)	16 (36.4)	19 (43.2)			
Diagnosis				119	−2.47	0.013^a^
Adenocarcinoma	23 (21.3)	42 (38.9)	43 (39.8)	108		
Smoking status				99	−1.944	0.052
Current or former	13 (32.5)	15 (37.5)	12 (30.0)			
Never	9 (15.3)	24 (40.7)	26 (44.1)			
Squamous cell carcinoma	7 (63.6)	2 (18.2)	2 (18.2)	11		
Smoking status				8	−0.77	0.643
Current or former	4 (66.7)	2 (33.3)	0 (0.0)			
Never	1 (50.0)	0 (0.0)	1 (50.0)			
Smoking status				111	−2.63	0.009^a^
Current or former	19 (38.0)	18 (36.0)	13 (26.0)			
Never	10 (16.4)	24 (39.3)	27 (44.3)			

Abbreviation: *PD-L1* programmed cell death ligand-1, *TPS* tumor proportion score; ^a^statistically significant

### IHC double staining with anti-TTF-1 and anti-PD-L1

**Fig. 2:**
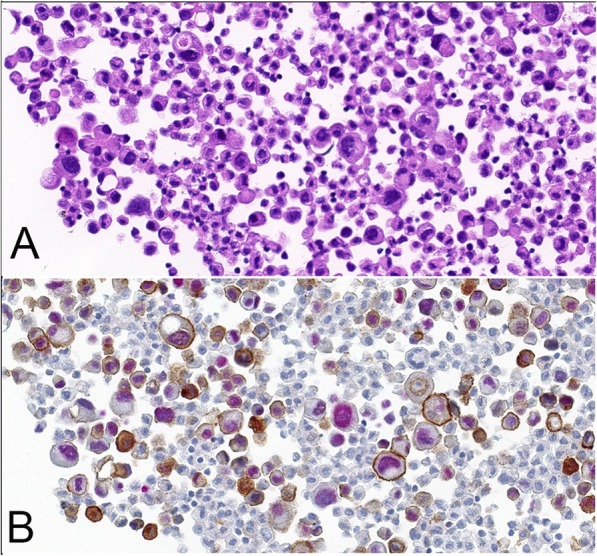
Immunocytochemical double staining with antibodies against TTF-1 (red) and PD-L1 (brown) in MPE cell block section (magnification × 40). **a** Hematoxylin-eosin. **b** Double staining easily distinguishes difficult-to-identify tumor cells from nonneoplastic cells

**Fig. 3:**
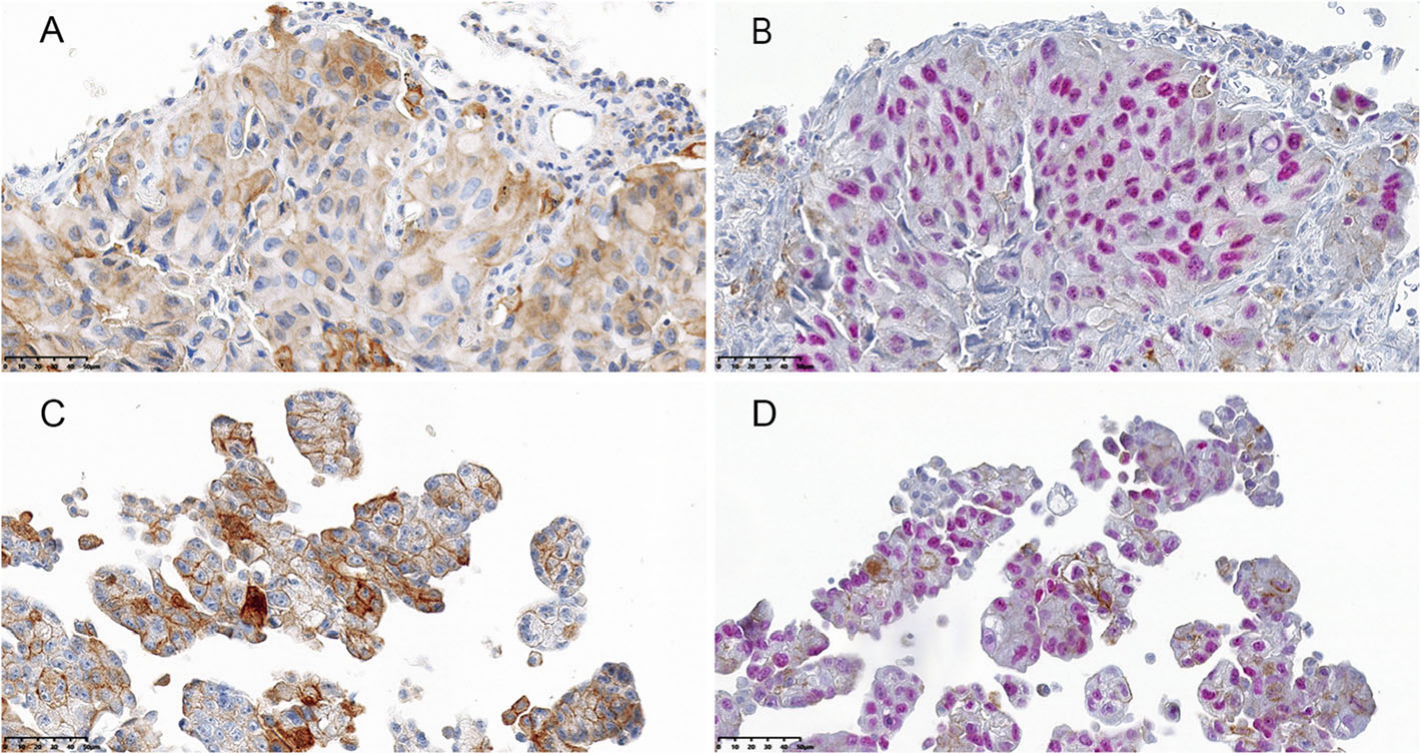
Discrepancy of tumor PD-L1 expression between immunohistochemical (IHC) single and double staining (magnification × 40). Single PD-L1 IHC staining (**a, c**) shows a higher tumor proportion score and stronger staining intensity compared with double IHC staining with anti-PD-L1 and TTF-1 (**b, d**) in both histology sample (**a** vs **b**) and MPE cell block (**c** vs **d**)

**Fig. 4:**
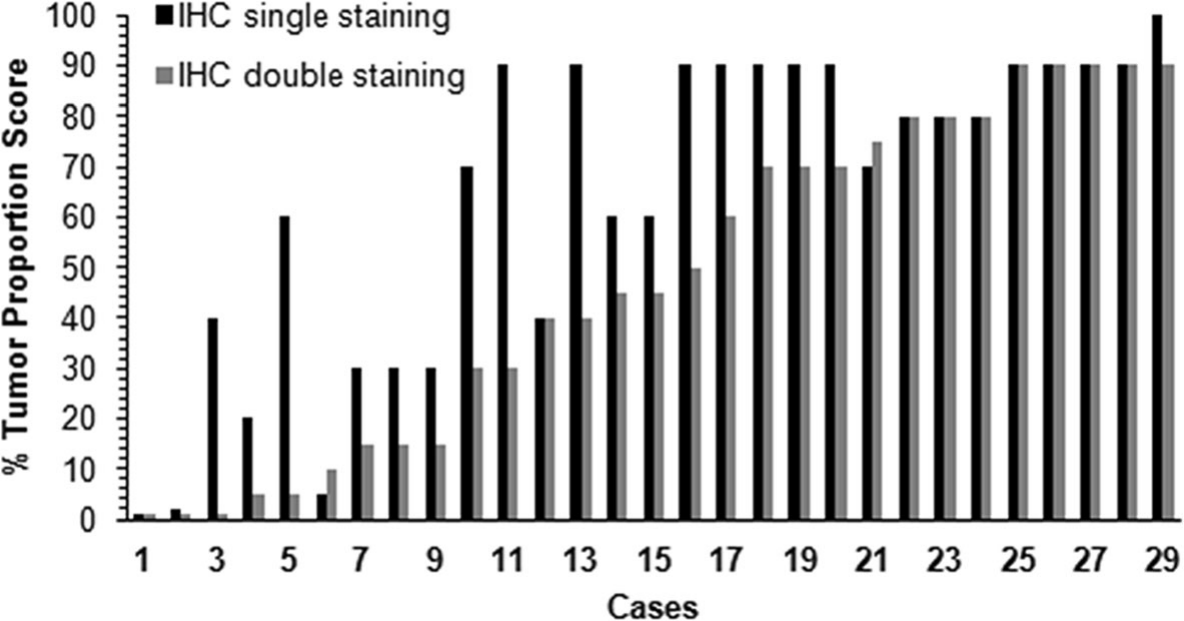
Comparison of tumor proportion scores (TPS) for PD-L1 expression between immunohistochemical (IHC) single and double staining. Double staining results in a lower TPS for PD-L1 expression compared with single staining. IHC single staining was performed using a VENTANA PD-L1 (SP263) Rabbit Monoclonal Primary Antibody assay. IHC double staining was performed using an automated Ventana IHC assay for TTF-1 (dilution 1:100; SPT24 clone, Leica, USA) with an ultraView Universal AP Red Detection Kit (Ventana Medical Systems, Tucson, AZ) on the basis of the IHC PD-L1 single-staining process

Twenty-nine of the 32 samples were subjected to IHC double staining with antibodies to TTF-1 and PD-L1. The remaining 3 cases were excluded for insufficient VTCs after the blocks were re-sectioned. As expected, IHC double staining allowed an easier analysis of IHC quantification of PD-L1 expression, especially when the malignant cells were distributed singly and interspersed with non-neoplastic cells (Fig. 2). Unfortunately, a portion of the double-stained cases showed weaker staining intensity (Fig. 3) and a low PD-L1 expression score (*p* = 0.000<0.05) compared with cases stained using the IHC single PD-L1 assay (Fig. 4).

## Discussion

The feasibility of using cytology samples for PD-L1 expression testing has been well reported [15, 20, 23, 25, 26]. However, to the best of our knowledge, the relevant data on the application of MPE cell block samples in PD-L1 testing are limited. And there is also no clear understanding of the specificity of this type of sample for PD-L1 detection. The results of the current study further confirmed the concordance between MPE cell blocks and matched histology samples in PD-L1 expression detection, which would certainly increase the confidence in the clinical application of MPE cell blocks to predict the efficacy of immunotherapy.

To better contrast our work with that in previous studies, we used three TPS cut-offs (< 1, 1–49% and ≥ 50%), which were used in the 22C3 assay, to evaluate PD-L1 expression. This decision was carefully considered and was based on the high concordance between the 22C3 and SP263 assays [14, 24, 27, 28]. In our study, the expression rate of PD-L1 with TPS ≥50% as a cut-off for either histology (29.7%) or cytology (37.7%) was similar to that noted in two previous studies of consecutive sample analyses [23, 25]. More importantly, we observed a significant difference in PD-L1 expression between MPE cell blocks and biopsy samples, which suggests that MPE cell block samples may be more suitable for PD-L1 detection than small biopsy samples.

The reason for the higher positive rate of PD-L1 expression that is often detected in cytology samples has remained unclear. Heterogeneity of tumor PD-L1 expression (including inter-tumoral and intra-tumoral) may explain this phenomenon [19, 29]. However, whether there are other potential causes is worth further exploring. We speculate that the suspended distribution of tumor cells in pleural fluid might be related to a higher PD-L1 positive rate of the MPE cell block. On the one hand, the dispersed suspension distribution may partially mitigate the effects of heterogeneity of tumor PD-L1 expression. However, due to the small size of the material, the results of PD-L1 expression obtained by evaluating small biopsies are significantly affected by intratumoral heterogeneity [30, 31]. The underestimation on the PD-L1 status of a whole tissue sample based on evaluating single biopsy from patient has been reported [32]. On the other hand, the distribution pattern of tumor cells with PD-L1 positivity has been described in previous literature, and it was found that these cells are more likely to be located at the tumor-stroma interface [19]. Single or tiny clusters of tumor cells suspended in pleural effusion may have more opportunities to interact with the immune microenvironment, in a similar manner as the tumor cells at the tumor-stroma interface. In fact, a previous study has suggested that there may be an immune interaction between pleural effusion tumor cells and macrophages [22]. Moreover, studies have confirmed that both macrophages and T cells in the tumor immune microenvironment can induce tumor cells to express PD-L1 through their distinct patterns [33, 34]. Therefore, it is reasonable to believe that PD-L1 expression in tumor cells in pleural effusion may be enhanced by an activated immune response.

To better clarify the possible reasons for the difference in PD-L1 expression between cytology and histology samples, we analyzed a number of relevant factors, including sample interval time, treatment differences, sampling types of histology samples, and the number of VTCs, but no significant correlations were found. The only difference before analysis was the fixation time in 10% NBF (15–30 min for MPE cell blocks vs 2–24 h for histology samples). Although the fixation method is indeed an important pre-analytical factor that affects the IHC results, [35, 36] in terms of PD-L1 detection, the current data indicated that different fixatives (formalin only vs methanol/alcohol only vs both) did not have significant effects on the evaluation of PD-L1 expression [18, 23]. Moreover, based on years of clinical practice, a 15-min fixation was considered to be sufficient for scattered single cells or tiny cell clusters because the cells were well mixed with 10% NBF in an oscillator. Nevertheless, the possible effects of fixation time should be further analyzed by more rigorous comparative tests. Furthermore, potential errors in the analytic and postanalytic phases were excluded by repeated testing and interpretation by multiple pathologists (for 15 pairs of inconsistent samples). The expression patterns of TTF-1 from IHC double-stained sections were also taken as references to exclude errors that could be caused by mistaking non-tumor cells for tumor cells.

Much research has reported the correlation between clinicopathological features and PD-L1 expression. However, no consensus has been reached. In our study, we found that patients with lung AC were more likely to have PD-L1-positive tumors than patients with SCC, which was consistent with the results of a recent meta-analysis [37] but different from a report on East Asian populations [38]. Since most of the samples included in this study were AC, we cannot exclude the influence of the small sample size of SCC on the reliability of the results. In addition, we found that for patients with advanced lung AC, positive smoking status was shown to be a negative factor for PD-L1 expression, which was different from the findings of some previous studies [22, 37, 39]. This contradiction may be related to the finding that self-reported smoking status does not accurately represent the presence of a molecular smoking signature [40].

Due to the cluster structure and malignant morphology of tumor cells, the evaluation of PD-L1 expression in MPE samples was not a problem for most cases. However, when these features were not common enough and tumor cells were interspersed with nontumor cells, the calculation of TPS was challenging. In this case, additional IHC stains must be utilized to identify tumor cells. To the best of our knowledge, no studies on the use of IHC double staining for PD-L1 detection have been published. We found only one study in which the author used double staining to detect PD-L1 and CD68, but they did not present relevant data [14]. Herein, we clarify the value of double staining in the interpretation of PD-L1 expression, but we also found that this method impaired PD-L1 expression detection compared with the results of a standard single staining process. We postulated that this might be related to the secondary antigen retrieval and multiple wash steps in the use of a second dye, which results in the elution of some of the protein-bound chromophores that had been bound to the tissues. We believe this can be resolved through further process optimization and calibration settings, which will be a focus of future studies.

As one of the limitations of the current study, small biopsy tissues accounted for the vast majority (91%) of the histology samples. The application of biopsy samples for tumor PD-L1 detection has its own limitations, which may affect the reliability of the results in the present study to some extent. Unfortunately, it isn’t feasible to compare the results between samples from surgical resection and MPE cell block as patients with advanced NSCLC have little chance to do surgery. In addition, the sections used for double staining were not obtained by continuous sectioning, which increased the impact of intratumoral heterogeneity on the results. Finally, this study was unable to further clarify the value of the clinical application of a relatively higher PD-L1 expression rate in MPE cell blocks due to a lack of relevant clinical treatment information, such as immunotherapy response rate and prognosis.

## Conclusions

In summary, our results demonstrate that MPE cell block samples are good candidates for PD-L1 expression detection in advanced NSCLC patients. The mechanism and clinical significance of the higher PD-L1 expression rate of MPE cell blocks compared with small biopsy samples should be evaluated prospectively.

## Data Availability

The datasets used or analyzed during the current study are available from the corresponding author on reasonable request.

## Abbreviations

PD-1: Programmed cell death protein-1
PD-L1: Programmed cell death ligand-1
NSCLC: Non-small cell lung carcinoma
IHC: Immunohistochemistry
MPE: Malignant pleural effusion
TTF-1: Thyroid transcription factor-1
H&E: Hematoxylin and eosin
TPS: Tumor proportion score
VTCs: Viable tumor cells
SIS: Staining intensity score
FNA: Fine needle aspiration
AC: Adenocarcinoma
SCC: Squamous cell carcinoma

## References

[1] Brahmer J, Reckamp KL, Baas P, Crino L, Eberhardt WE, Poddubskaya E, Antonia S, Pluzanski A, Vokes EE, Holgado E (2015). Nivolumab versus Docetaxel in advanced squamous-cell non-Small-cell lung Cancer. N Engl J Med.

[2] Herbst RS, Baas P, Kim DW, Felip E, Perez-Gracia JL, Han JY, Molina J, Kim JH, Arvis CD, Ahn MJ (2016). Pembrolizumab versus docetaxel for previously treated, PD-L1-positive, advanced non-small-cell lung cancer (KEYNOTE-010): a randomised controlled trial. Lancet.

[3] Garon EB, Rizvi NA, Hui R, Leighl N, Balmanoukian AS, Eder JP, Patnaik A, Aggarwal C, Gubens M, Horn L (2015). Pembrolizumab for the treatment of non–Small-cell lung Cancer. N Engl J Med.

[4] Eroglu Z, Zaretsky JM, Hu-Lieskovan S, Kim DW, Algazi A, Johnson DB, Liniker E, Ben K, Munhoz R, Rapisuwon S (2018). High response rate to PD-1 blockade in desmoplastic melanomas. Nature.

[5] Topalian SL, Sznol M, McDermott DF, Kluger HM, Carvajal RD, Sharfman WH, Brahmer JR, Lawrence DP, Atkins MB, Powderly JD (2014). Survival, durable tumor remission, and Long-term safety in patients with advanced melanoma receiving Nivolumab. J Clin Oncol.

[6] Zhao B, Zhang W, Yu D, Xu J, Wei Y (2018). The benefit and risk of nivolumab in non-small-cell lung cancer: a single-arm meta-analysis of noncomparative clinical studies and randomized controlled trials. Cancer Med.

[7] Knorr DA, Ravetch JV (2019). Immunotherapy and Hyperprogression: unwanted outcomes, Unclear Mechanism. Clin Cancer Res.

[8] Borcoman E, Kanjanapan Y, Champiat S, Kato S, Servois V, Kurzrock R, Goel S, Bedard P, Le Tourneau C (2019). Novel patterns of response under immunotherapy. Ann Oncol.

[9] Ferrara R, Mezquita L, Texier M, Lahmar J, Audigier-Valette C, Tessonnier L, Mazieres J, Zalcman G, Brosseau S, Le Moulec S (2018). Hyperprogressive disease in patients with advanced non-Small cell lung Cancer treated with PD-1/PD-L1 inhibitors or with single-agent chemotherapy. JAMA Oncol.

[10] Zhou J, Yao H, Zhao J, Zhang S, You Q, Sun K, Zou Y, Zhou C, Zhou J (2015). Cell block samples from malignant pleural effusion might be valid alternative samples for anaplastic lymphoma kinase detection in patients with advanced non-small-cell lung cancer. Histopathology.

[11] Wang W, Tang Y, Li J, Jiang L, Jiang Y, Su X (2015). Detection of ALK rearrangements in malignant pleural effusion cell blocks from patients with advanced non-small cell lung cancer: a comparison of Ventana immunohistochemistry and fluorescence in situ hybridization. Cancer Cytopathol.

[12] Munari E, Zamboni G, Sighele G, Marconi M, Sommaggio M, Lunardi G, Rossi G, Cavazza A, Moretta F, Gilioli E (2019). Expression of programmed cell death ligand 1 in non-small cell lung cancer: comparison between cytologic smears, core biopsies, and whole sections using the SP263 assay. Cancer Cytopathol.

[13] Xu H, Bratton L, Nead M, Russell D, Zhou Z (2018). Comparison of programmed death-ligand 1 (PD-L1) immunostain for nonsmall cell lung carcinoma between paired cytological and surgical specimens. Cytojournal.

[14] Capizzi E, Ricci C, Giunchi F, Zagnoni S, Ceccarelli C, Gomez BUA, Casolari L, Gelsomino F, Trisolini R, Fiorentino M (2018). Validation of the immunohistochemical expression of programmed death ligand 1 (PD-L1) on cytological smears in advanced non small cell lung cancer. Lung Cancer.

[15] Ilie M, Juco J, Huang L, Hofman V, Khambata-Ford S, Hofman P (2018). Use of the 22C3 anti-programmed death-ligand 1 antibody to determine programmed death-ligand 1 expression in cytology samples obtained from non-small cell lung cancer patients. Cancer Cytopathol.

[16] Bashover E, Arriola AG, Joseph CT, Staerkel G, Wang WB, Roy-Chowdhuri S (2019). The use of cytological material in melanoma for programmed death ligand 1 immunostaining. Cytopathology.

[17] Jain D, Sukumar S, Mohan A, Iyer VK (2018). Programmed death-ligand 1 immunoexpression in matched biopsy and liquid-based cytology samples of advanced stage non-small cell lung carcinomas. Cytopathology.

[18] Noll B, Wang WL, Gong Y, Zhao J, Kalhor N, Prieto V, Staerkel G, Roy-Chowdhuri S (2018). Programmed death ligand 1 testing in non-small cell lung carcinoma cytology cell block and aspirate smear preparations. Cancer Cytopathol.

[19] McLaughlin J, Han G, Schalper KA, Carvajal-Hausdorf D, Pelekanou V, Rehman J, Velcheti V, Herbst R, LoRusso P, Rimm DL (2016). Quantitative assessment of the heterogeneity of PD-L1 expression in non-Small-cell lung Cancer. JAMA Oncol.

[20] Russell-Goldman E, Kravets S, Dahlberg SE, Sholl LM, Vivero M (2018). Cytologic-histologic correlation of programmed death-ligand 1 immunohistochemistry in lung carcinomas. Cancer Cytopathol.

[21] Mansour MSI, Seidal T, Mager U, Baigi A, Dobra K, Dejmek A (2017). Determination of PD-L1 expression in effusions from mesothelioma by immuno-cytochemical staining. Cancer.

[22] Tseng YH, Ho HL, Lai CR, Luo YH, Tseng YC, Whang-Peng J, Lin YH, Chou TY, Chen YM (2018). PD-L1 expression of tumor cells, macrophages, and immune cells in non-Small cell lung Cancer patients with malignant pleural effusion. J Thorac Oncol.

[23] Wang H, Agulnik J, Kasymjanova G, Wang A, Cohen V, Small D, Pepe C, Sakr L, Fiset P, Auger M (2018). Cytology cell blocks are suitable for immunohistochemical testing for PD-L1 in lung cancer. Ann Oncol.

[24] Humphries MP, McQuaid S, Craig SG, Bingham V, Maxwell P, Maurya M, McLean F, Sampson J, Higgins P, Greene C (2019). Critical appraisal of programmed death ligand 1 reflex diagnostic testing: current standards and future opportunities. J Thorac Oncol.

[25] Heymann JJ, Bulman WA, Swinarski D, Pagan CA, Crapanzano JP, Haghighi M, Fazlollahi L, Stoopler MB, Sonett JR, Sacher AG (2017). Programmed death-ligand 1 expression in non-small cell lung carcinoma: comparison among cytology, small biopsy, and surgical resection specimens. Cancer.

[26] Skov BG, Skov T (2017). Paired comparison of PD-L1 expression on Cytologic and histologic specimens from malignancies in the lung assessed with PD-L1 IHC 28-8pharmDx and PD-L1 IHC 22C3pharmDx. Appl Immunohistochem Mol Morphol.

[27] Buttner R, Gosney JR, Skov BG, Adam J, Motoi N, Bloom KJ, Dietel M, Longshore JW, Lopez-Rios F, Penault-Llorca F (2017). Programmed death-ligand 1 immunohistochemistry testing: a review of analytical assays and clinical implementation in non-Small-cell lung Cancer. J Clin Oncol.

[28] Tsao MS, Kerr KM, Kockx M, Beasley MB, Borczuk AC, Botling J, Bubendorf L, Chirieac L, Chen G, Chou TY (2018). PD-L1 immunohistochemistry comparability study in real-life clinical samples: results of blueprint phase 2 project. J Thorac Oncol.

[29] Niemeijer AN, Leung D, Huisman MC, Bahce I, Hoekstra OS, van Dongen G, Boellaard R, Du S, Hayes W, Smith R (2018). Whole body PD-1 and PD-L1 positron emission tomography in patients with non-small-cell lung cancer. Nat Commun.

[30] Munari E, Zamboni G, Marconi M, Sommaggio M, Brunelli M, Martignoni G, Netto GJ, Moretta F, Mingari MC, Salgarello M (2017). PD-L1 expression heterogeneity in non-small cell lung cancer: evaluation of small biopsies reliability. Oncotarget.

[31] Haragan A, Field JK, Davies MPA, Escriu C, Gruver A, Gosney JR (2019). Heterogeneity of PD-L1 expression in non-small cell lung cancer: implications for specimen sampling in predicting treatment response. Lung Cancer.

[32] Ilie M, Long-Mira E, Bence C, Butori C, Lassalle S, Bouhlel L, Fazzalari L, Zahaf K, Lalvee S, Washetine K (2016). Comparative study of the PD-L1 status between surgically resected specimens and matched biopsies of NSCLC patients reveal major discordances: a potential issue for anti-PD-L1 therapeutic strategies. Ann Oncol.

[33] DeNardo DG, Ruffell B (2019). Macrophages as regulators of tumour immunity and immunotherapy. Nat Rev Immunol.

[34] Wei Y, Zhao Q, Gao Z, Lao XM, Lin WM, Chen DP, Mu M, Huang CX, Liu ZY, Li B (2019). The local immune landscape determines tumor PD-L1 heterogeneity and sensitivity to therapy. J Clin Invest.

[35] Kerr KM, Bubendorf L, Edelman MJ, Marchetti A, Mok T, Novello S, O'Byrne K, Stahel R, Peters S, Felip E (2014). Second ESMO consensus conference on lung cancer: pathology and molecular biomarkers for non-small-cell lung cancer. Ann Oncol.

[36] Thunnissen E, Kerr KM, Herth FJ, Lantuejoul S, Papotti M, Rintoul RC, Rossi G, Skov BG, Weynand B, Bubendorf L (2012). The challenge of NSCLC diagnosis and predictive analysis on small samples. Practical approach of a working group. Lung Cancer.

[37] Petrelli F, Maltese M, Tomasello G, Conti B, Borgonovo K, Cabiddu M, Ghilardi M, Ghidini M, Passalacqua R, Barni S (2018). Clinical and molecular predictors of PD-L1 expression in non-Small-cell lung Cancer: systematic review and meta-analysis. Clin Lung Cancer.

[38] Pan Y, Zheng D, Li Y, Cai X, Zheng Z, Jin Y, Hu H, Cheng C, Shen L, Wang J (2017). Unique distribution of programmed death ligand 1 (PD-L1) expression in east Asian non-small cell lung cancer. J Thorac Dis.

[39] Kadara H, Choi M, Zhang J, Parra ER, Rodriguez-Canales J, Gaffney SG, Zhao Z, Behrens C, Fujimoto J, Chow C (2017). Whole-exome sequencing and immune profiling of early-stage lung adenocarcinoma with fully annotated clinical follow-up. Ann Oncol.

[40] Rizvi NA, Hellmann MD, Snyder A, Kvistborg P, Makarov V, Havel JJ, Lee W, Yuan J, Wong P, Ho TS (2015). Cancer immunology. Mutational landscape determines sensitivity to PD-1 blockade in non-small cell lung cancer. Science.

